# Development of Peptide-Based Nanoparticles for Mitochondrial Plasmid DNA Delivery

**DOI:** 10.3390/polym13111836

**Published:** 2021-06-01

**Authors:** Rúben Faria, Eric Vivés, Prisca Boisguerin, Angela Sousa, Diana Costa

**Affiliations:** 1 CICS-UBI—Health Sciences Research Centre, University of Beira Interior, Av. Infante D. Henrique, 6200-506 Covilhã, Portugal; ruben_faria95@hotmail.com (R.F.); angela@fcsaude.ubi.pt (A.S.); 2 PhyMedExp, Université de Montpellier, INSERM, CNRS, 34295 Montpellier, France; eric.vives@umontpellier.fr (E.V.); prisca.boisguerin@crbm.cnrs.fr (P.B.)

**Keywords:** biocompatibility, cell-penetrating peptides, mitochondrial DNA diseases, mitochondria targeting, nano-delivery systems, plasmid DNA

## Abstract

A mitochondrion is a cellular organelle able to produce cellular energy in the form of adenosine triphosphate (ATP). As in the nucleus, mitochondria contain their own genome: the mitochondrial DNA (mtDNA). This genome is particularly susceptible to mutations that are at the basis of a multitude of disorders, especially those affecting the heart, the central nervous system and muscles. Conventional clinical practice applied to mitochondrial diseases is very limited and ineffective; a clear need for innovative therapies is demonstrated. Gene therapy seems to be a promising approach. The use of mitochondrial DNA as a therapeutic, optimized by peptide-based complexes with mitochondrial targeting, can be seen as a powerful tool in the reestablishment of normal mitochondrial function. In line with this requirement, in this work and for the first time, a mitochondrial-targeting sequence (MTS) has been incorporated into previously researched peptides, to confer on them a targeting ability. These peptides were then considered to complex a plasmid DNA (pDNA) which contains the mitochondrial gene ND1 (mitochondrially encoded NADH dehydrogenase 1 protein), aiming at the formation of peptide-based nanoparticles. Currently, the ND1 plasmid is one of the most advanced bioengineered vectors for conducting research on mitochondrial gene expression. The formed complexes were characterized in terms of pDNA complexation capacity, morphology, size, surface charge and cytotoxic profile. These data revealed that the developed carriers possess suitable properties for pDNA delivery. Furthermore, in vitro studies illustrated the mitochondrial targeting ability of the novel peptide/pDNA complexes. A comparison between the different complexes revealed the most promising ones that complex pDNA and target mitochondria. This may contribute to the optimization of peptide-based non-viral systems to target mitochondria, instigating progress in mitochondrial gene therapy.

## 1. Introduction

Mitochondria are cytoplasmic organelles responsible for energy production to ensure normal cell metabolic function. This organelle synthesizes ATP via oxidative phosphorylation, in which four enzymatic systems are involved to promote electron transport and generate a proton gradient [1,2]. Mitochondria are also involved in cellular signaling, ion homeostasis, and the metabolism of amino acids, lipids, cholesterol, steroids and nucleotides [3,4]. Beyond this, its role in the regulation of the cell cycle, cell growth and apoptosis is also reported [5,6]. Along with chloroplasts, mitochondria emerged from bacterial ancestors, and through evolution, retained specialized structures and haploid genomes [7]. Much of their genetic content is included in the nuclear genome, but a significant part remains in mitochondria. The mitochondrial genome, mtDNA, is a double-stranded molecule, 16 kbp in size, containing 37 genes that encode 13 polypeptides that take part in the oxidative phosphorylation chain, 2 rRNAs and 22 tRNAs, all exclusive to the mitochondria [8]. Mutations in mtDNA have been associated with multiple metabolic and neuromuscular degenerative syndromes, and connected with Parkinson’s and Alzheimer’s diseases, diabetes and several types of cancer [9,10,11,12,13,14,15,16,17]. The therapeutic approaches to treat mtDNA diseases have been largely ineffective, as they focus on alleviation of the symptoms. In this sense, mitochondrial gene therapy can be seen as a valuable and powerful tool to deal with these disorders, as it fights the disease at its source [18,19,20]. The direct and efficient transfection of mitochondria, with regard to replacing mutated mitochondrial genes, requires a suitable delivery system. This vehicle should protect, carry and target the genetic content to mitochondria, promoting its efficient release, and thus, ensure mitochondrial gene and protein expression.

In the last decades, considerable advances have been made in the conception of nucleic acid-based delivery systems to overcome the major drawbacks of payload delivery to eukaryotic cells, namely, cellular uptake/internalization, endosomal escape, targeting a specific subcellular compartment, and ultimately, the induction of therapeutic action [19,21,22,23,24]. In line with this aim, micelles, polymers, lipid- and peptide-based nanoparticles are among the most studied systems for gene release [25,26,27,28]. In particular, cell-penetrating peptides (CPP) offer exceptional properties to be explored as gene delivery vehicles for successful gene therapy [28,29,30,31,32]. CPPs are short peptides, usually of fewer than 30 amino acids, and can be separated into arginine-rich and amphipathic peptides. The amphipathic ones possess both hydrophilic and hydrophobic domains that confer on these peptides the ability to interact with the genetic material, promoting its membrane translocation, followed by cell entry [28,30,33]. However, the exact mechanism CPPs use to penetrate cells is still not fully understood, with endocytosis and direct penetration being the most probable hypothesis [30,33]. Despite this fact, their great potential in conjugation with therapeutic molecules and in their cellular internalization has been well recognized, leading to the significant participation of CCPs in biomedical applications [31,32,33,34,35,36].

In this work, a set of CPPs have been designed/synthesized to complex the mitochondrial gene ND1-encoded pDNA (pND1) and ensure its targeting of mitochondria. To the best of our knowledge, to date, this pND1 is by far one of the most advanced bioengineered vectors in research toward mitochondrial gene expression. It enables us to get close to the concrete reality of mitochondrial protein expression. The formed nano-complexes were developed at various nitrogen-to-phosphate group (N/P) ratios and adequately characterized. Their biocompatibility has been assessed, and in vitro studies have been performed to evaluate the ability of the developed peptide/pND1 complexes to target mitochondria. The suitable physicochemical properties of the novel delivery systems, along with their mitochondria-targeting capacity, make them useful nano-platforms for mitochondrial gene therapy. Furthermore, a comparison between the various peptide/pND1 complexes revealed differences in their physicochemical properties and showed the ones with high potential for mitochondria targeting. This work may contribute to optimizing the conception of novel peptide-based systems for long-term mitochondrial gene expression.

## 2. Materials and Methods

### 2.1. Materials

Fmoc-amino acids, dimethylformamide (DMF), trifluoroacetic acid (TFA), diisopropylcarbodiimide (DIC), diisopropylethylamine (DIEA), dichloromethane (DCM), piperidine, oxyma, diethyl ether, acetonitrile and fluorescein isothiocyanate (FITC) were all obtained from Sigma-Aldrich (St Loius, MO, USA). Resin for peptide synthesis (AmphiSpheres 40™) was obtained from Agilent Technologies (Les Ulis, France). The Liberty Blue HT12™ Automated Microwave Peptide Synthesizer was obtained from CEM (NC, USA). The FC 204 Fraction Collector and 321 HPLC Pumps were obtained from Gilson. The Pharmacia LKB-REC 102 was obtained from Pharmacia (Stockholm, Sweden). The Waters Alliance 2695 HPLC System was obtained from Waters Corporation. DAPI was from Invitrogen (Carlsbad, CA, USA) and MitoTracker Orange CMTMRos from Molecular Probes (Leiden, The Netherlands). All solutions were freshly prepared using ultra-pure water, purified with a Milli-Q system from Millipore (Billerica, MA, USA).

Normal Human Dermal Fibroblasts (NHDF), Ref. C-12302 (cryopreserved cells), and cancer HeLa cells were purchased from PromoCell (Heidelberg, Germany) and Invitrogen (Carlsbad, CA, USA), respectively.

The plasmid pCAG-GFP-ND1 (5.4 kbp) was developed by our research group through the cloning of the mitochondrial NADH dehydrogenase 1 protein-encoded gene (mtND1) in *Escherichia coli*. The full description of gene cloning and plasmid production are described elsewhere [19].

### 2.2. Methods

#### 2.2.1. Synthesis of Peptides

Peptide synthesis was performed using the Liberty Blue HT12™ Automated Microwave Peptide Synthesizer. The peptides were produced by following the Fmoc approach of solid-phase peptide synthesis (SPPS) methodology. Amino acid-COOH activation was performed using a 1M solution of DIC (39.4 mL in 250 mL of DMF) and oxyma + DIEA (17.76 g + 6.25 mL in 125 mL of DMF). After the addition of amino acid, the Fmoc group was removed using a 20% solution of piperidine in DMF. The peptides were deprotected and cleaved from their respective resins by TFA treatment (TFA 92.5%, triisopropylsilane 5%, and water 2.5%). Peptides were purified by semi-preparative HPLC (Waters Corp., Wilmslow, UK), using an octadodecyl carbon chain (C18) column. The tryptophan-(W) and arginine-(R) rich amphipatic peptides (WRAP) WRAP1, WRAP5 and MTS peptides were dissolved in H_2_O + 0.1% TFA, and MTS-WRAP1, MTS-WRAP5, (KH)9, MTS-(KH)9 and CpMTP were resuspended in H_2_O + 30% acetonitrile. The purification of all peptides was carried out using the same gradient (20% to 60% of acetonitrile for 40 min at a flow rate of 5 mL/min). LC/MS analysis was performed to assess the molecular mass of each peptide, confirming a purity of ≥95%.

#### 2.2.2. Formulation of Peptide/pND1 Complexes

pND1 stock solution was prepared in sodium acetate buffer (0.1 mM sodium acetate/0.1 M acetic acid, pH 4.5). Peptide/pND1 complexes were formed at different N/P ratios. The calculation of the N/P ratio is defined as the molar relation of amine groups in the peptide, which represent the positive charges, to phosphate groups in the pND1, which represent the negative charges, considering the mass per charge ratio of pND1 (330 g/mol, relative to one phosphate group) [19].

Therefore, for the preparation of peptide/pND1 complexes at various N/P ratios, different concentrations of each peptide (50 µL) were added to pND1 solution at a fixed concentration of 1.4 nM. The mixture was vortexed for 30 s and left for equilibration for 25 min at room temperature. The complexes were then centrifuged at 13,000× *g* for 20 min at 4 °C and the pellet, containing the pND1-based nanoparticles, was recovered.

The presence of pND1 in the supernatant was evaluated by the horizontal electrophoresis technique for 30 min under 120 V in 1% agarose gel stained with GreenSafe Premium (NZYTech, Lda. Lisbon, Portugal). The gels were visualized using the Gel documentation system under UV light (UVItec Limited, Cambridge, UK).

#### 2.2.3. Scanning Electron Microscopy (SEM)

The morphology of peptide/pND1 complexes was investigated by scanning electron microscopy (SEM). Freshly prepared complexes were washed three times with 300 µL of ultra-pure grade water and centrifuged (13,000× *g*, 12 min, 4 °C). Then, the pellet was recovered and resuspended in 40 µL of 2% tungsten solution. The samples were diluted 1:20 in ultra-pure water, 10 µL pipetted to the roundly shaped coverslip (10 mm) and left to dry overnight at room temperature. On the following day, the samples were mounted on aluminum supports, fixed with double-sided adhesive tape and sputter coated with gold using an Emitech K550 (London, UK) sputter coater. A scanning electron microscope, Hitachi S-2700 (Tokyo, Japan), with accelerating voltage of 20 kV at various magnifications, was used to determine the morphology of peptide/pND1 complexes.

#### 2.2.4. Particle Size and Zeta Potential Measurements

The average particle size and the zeta potential of peptide/pND1 complexes were determined by dynamic light scattering (DLS) using a Zetasizer Nano ZS (Malvern Instruments, Worcestershire, UK). The pellet containing the complexes was suspended in 5% glucose with 1 mM NaCl. DLS using a He-Ne laser 633 nm with non-invasive backscatter optics (NIBS) and electrophoretic light scattering using M3-PALS laser technique (phase analysis light scattering) were applied for size and charge determination, respectively. The Malvern zetasizer software v 6.34 (Worcestershire, UK) was used.

#### 2.2.5. Cell Culture

Cancer HeLa cells and fibroblast cells were grown in Dulbecco´s Modified Eagle´s Medium, high glucose (DMEM-HG) (Sigma-Aldrich, St. Loius, MO, USA) supplemented with 10% heat-inactivated bovine fetal serum (FBS), 0.5 g/L sodium bicarbonate, 1.10 g/L HEPES, 100 μg/mL of streptomycin and 100 units/mL of penicillin (Sigma-Aldrich, St. Loius, MO, USA). The cells were kept at 37 °C in a 5% CO_2_ humidified atmosphere until confluence was attained.

#### 2.2.6. Cytotoxicity Evaluation

The biocompatibility of the systems was evaluated on HeLa cells by means of MTT (3-[4,5dimethyl-thiazol-2-yl]-2,5-diphenyltetrazolium bromide) assay. MTT assay is a colorimetric method that quantifies the metabolic active cells. HeLa cells were plated in a 96-well plate, at a density of 1 × 10^4^ cells/well, and grown at 37 °C in a 95% O_2_/5% CO_2_ humidified atmosphere. The complexes (50 µL), in serum-free DMEM medium, were applied to the well plates. After 24 h or 48 h incubation, the redox activity was assessed through the reduction of MTT. Absorbance at 570 nm was measured using a Biorad Microplate Reader Benchmark. The spectrophotometer was calibrated to zero absorbance using the culture medium without cells. Non-transfected cells were used as negative control and ethanol-treated cells were used as positive control. The relative cell viability (%) related to control wells was calculated by [A]test/[A]control × 100, where [A] test is the absorbance of the test sample and [A] control is the absorbance of the negative control sample. All the experiments were repeated three times in triplicate.

#### 2.2.7. Detection of Associated/Internalized pND1

pND1 was labeled with FITC by mixing 5 μL of pDNA, 73 μL of labeling buffer (0.1 M Sodium Tetraborate, pH 8.5) and 2 μL of FITC (in sterile anhydrous dimethyl sulfoxide, 500 mg/mL). The samples were placed under constant stirring for 4 h at room temperature and protected from light. One volume of 3 M sodium chloride (85 µL) and 2.5 volumes of 100% ethanol (212.5 µL) were added, and samples with the stained pND1 were incubated overnight at −20 °C. Thereafter, the solution was centrifuged at 12,000× *g* for 30 min at 4 °C, and the pellet was washed with 75% ethanol.

Cancer HeLa or fibroblast cells were cultured as described, and for transfection, 100 μL of peptides/FITC-pND1 complexes were added to each well. After 12 h, cells were washed twice with PBS and pND1 levels were estimated by measuring FITC fluorescence levels using a fluorimeter plate reader (excitation and emission wavelengths at 495 nm and 525 nm, respectively), based on a standard curve for FITC fluorescence.

#### 2.2.8. Cellular Organelle-Associated Fluorescence

The mitochondrial targeting capacity of the developed peptide/pND1 complexes has been evaluated by monitoring the associated FITC-pND1 fluorescence. Transfection has been mediated in HeLa or fibroblast cells by the peptide/pND1 complexes, as described before, and thereafter mitochondria and cytosol have been separated using the Mitochondria Isolation Kit for Cultured Cells (#89874, Thermo Fisher Scientific Inc., Rockford, IL, USA). This method leads to the efficient isolation of mitochondria with optimized purity and high/consistent yield [18] HeLa cells (1 × 10^4^) or fibroblasts (2 × 10^4^) have been transferred to Falcon tubes and 800 µL of mitochondria isolation reagent A was added to the cells, followed by incubation on ice for 2 min. Afterward, mitochondria isolation reagent B (10 µL) was added and HeLa or fibroblast cells were vortexed at maximum speed for 10 sec, incubated on ice for 5 min, and vortexed again at maximum speed every minute. At this point, 800 µL of Reagent C was then added, the samples were centrifuged at 800,000× *g* for 10 min at 4 °C and the supernatant was centrifuged at 3000× *g* for 10 min at 4 °C. Following this procedure, the obtained pellet contains the mitochondria, while the supernatant contains the cytosolic fraction. Reagent C (500 µL) was added to the pellet, a new centrifugation at 12,000× *g* for 5 min was performed, and the supernatant was discarded. The pellets consisting of mitochondria were resuspended in 50 µL of ice-cold PBS, mixed with 500 µL of carbonate buffer (fresh cold 0.1 M Na_2_CO_3_) and used in the FITC-pND1 fluorescence quantification experiments.

#### 2.2.9. Fluorescence Confocal Microscopy

##### Live Cell Imaging

The cellular uptake and mitochondria targeting ability of peptide/pND1 complexes were evaluated by confocal laser scanning microscopy (CLSM). FITC-labeled pDNA was complexed with the different peptides to form the nanoparticles, as described before. HeLa cells (2 × 10^3^) were grown in μ-slide 8-well until 50–60% confluence was achieved. The nucleus was marked with DAPI, and the mitochondria with MitoTracker Orange dye. The complete medium was replaced by a serum-free culture medium 12 h before transfection. Labeled FITC at 1 µg pND1 were added to each well. The images of HeLa cells transfected with the different peptide/pDNA complexes were acquired. Real live transfection was visualized using LSM 710 confocal microscope (Carl Zeiss SMT, Inc., Oberkochen, Germany) under a 63× oil immersion objective and analyzed with the LSM software (Carl Zeiss SMT, Inc., Oberkochen, Germany). During the experiment, HeLa cells were maintained at 37 °C with 5% CO_2_. All images were acquired with the laser and the filters corresponding to the respective DAPI (445/450 nm), FITC (525/550 nm) and MitoTracker (555/580 nm) dyes.

#### 2.2.10. Statistical Analysis

Normality tests (D´Agostino & Pearson omnibus and Kolmogorov–Smirnov) were applied to determine the normality of distribution of the sample data. One-way or two-way analysis of variance (ANOVA), with the Bonferroni test, was used for comparing the data of control and multiple experimental groups. A confidence interval of 95% (*p* < 0.05) was considered statistically significant. Data analysis was performed with GraphPad Prism v.8.01 (GraphPad Software, Inc., San Diego, CA, USA).

## 3. Results and Discussion

### 3.1. Synthesis of Peptides

CPPs have been demonstrated to facilitate payload delivery into the cells, especially by forming nanoparticles. In this study, we were interested in the synthesis of peptides with mitochondria-targeting ability to promote the targeting of peptide/pND1 nanoparticles to this organelle. This is considered a crucial step in the development of a suitable delivery system for mitochondrial gene therapy implementation. To accomplish this, we took advantage of the previously developed family of short tryptophan-(W) and arginine-(R) rich amphipathic peptides (WRAP) [29,30,31]. WRAP peptides demonstrated the ability to easily and efficiently encapsulate nucleic acids, including siRNA [30] and pDNA [29], leading to the conception of stable nano-systems. This asset has been explored to promote the delivery of genetic material to different cell types; this also contributes to the fact that WRAP-based nanoparticles enter cells mainly via direct translocation [31]. The peptide (KH)9 consists of a lysine–histidine repeat with high cationic charge density, a property that favors its penetration into cells. The amino acid lysine promotes the condensation of nucleic acids, facilitating their cellular uptake, while histidine plays a crucial role in avoiding endosomal sequestration, due to the proton sponge effect, allowing nucleic acids to reach the cytosol [37]. Despite the convenient properties, displayed by the mentioned peptides for cell uptake and gene delivery, none of the peptides exhibit mitochondrial affinity. Therefore, in this work, MTS was incorporated into each peptide. The chosen sequence consists of a 12-residue partial pre-sequence of yeast cytochrome c oxidase subunit IV, known to grant mitochondrial affinity to CPPs. The addition of MTS to WRAP1, WRAP5 and (KH)9 allowed the creation of multifunctional dual-domain peptides, capable of formulating nanoparticles with a hydrophilic core where pDNA was condensed and MTS located mostly on the surface, providing targeting to mitochondria [38]. The CpMTP peptide, which was synthesized based on the signal sequence of human mitochondrial methionine-R-sulfoxide reductase B2, has a natural affinity for mitochondria [39]. This peptide has the ability to condense and deliver high molecular weight cargo molecules, and to bind pDNA.

After synthesis, all peptides were purified by HPLC to obtain >95% purity and their mass and sequences were confirmed by mass spectrometry. The properties of the peptides, namely, sequence, total residues, isotopic mass, and positive charges, are presented in Table 1.

### 3.2. pND1 Complexation Capacity

**Figure 1:**
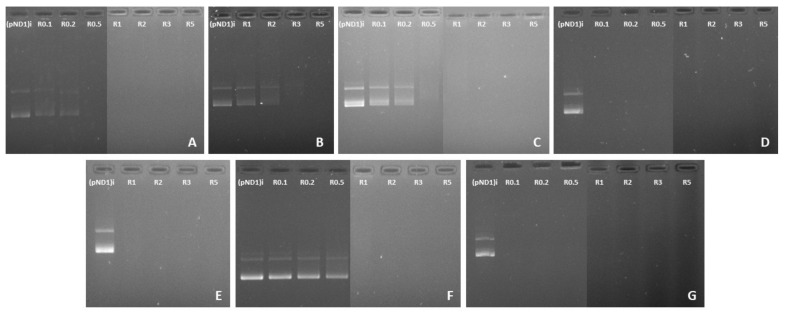
Analysis of pND1 complexation capacity of CpMTP/pND1 (**A**); WRAP1/pND1 (**B**); WRAP5/pND1 (**C**); (KH)9/pND1 (**D**); MTS-WRAP1/pND1 (**E**); MTS-WRAP5/pND1 (**F**) and MTS-(KH)9/pND1 (**G**) complexes at various N/P ratios, investigated by agarose gel electrophoresis. The samples were loaded at the application site at the upper end of the image (anode) and migrated then to the lower end (cathode).

In the last decade, there has been a crescendo of interest in the development of suitable nano-delivery systems to carry genetic content into the proximity of mitochondria and ensure its long-term expression, a strategy to deal with diseases related to mtDNA mutations [19,20,40,41,42,43,44]. In this work, peptide/pND1 complexes have been developed aiming to target mitochondria and deliver pND1 plasmid into this organelle, as the first step for efficient mitochondrial gene expression. In addition, our work also aimed to perform a comparison study to reveal the most promising peptide/pND1 complexes for subsequent studies on mitochondrial gene/protein expression. To accomplish this, MTS-peptide/pND1 and CpMTP/pND1 complexes were formed by a co-precipitation method—an approach used by other authors to encapsulate DNA and form DNA-based nanoparticles [19,40,44]. For comparison purposes and to unravel the role of the MTS sequence, WRAP1-, WRAP5- and (KH)9 peptides/pND1 complexes were also formulated. Each peptide interacted, mainly by electrostatic forces, with the negatively charged pND1 forming nano-sized particles. The extent of this interaction and, therefore, the capacity to complex pND1, may vary with the peptide and strongly depends on the N/P ratio considered at the complexes formulation step. The pND1 degree of complexation for the different peptide/pND1 systems and at various N/P ratios has been investigated by agarose gel electrophoresis. The results are shown in Figure 1. For each peptide, a screening study revealed the range of N/P ratios adequate to promote an efficient pND1 complexation. Although the study demonstrated that all peptides were able to complex pND1, differences arose concerning the N/P ratio required to ensure an efficient plasmid complexation. As can be observed in Figure 1A, the CpMTP peptide induced pND1 complexation from an N/P ratio of 0.5. From this N/P, the peptide efficiently neutralized the charges of pND1 and, thus, could not migrate through the agarose gel. As the N/P ratio further increases, it is expected that the positive charges from the amines strengthen the interaction between CpMTP and pND1, increasing the complexation degree of the latter molecule. Figure 1B demonstrated that WRAP1 started to induce pND1 complexation at a higher N/P ratio (3). When WRAP5 was considered to complex pND1, a lower N/P ratio (0.5), the same ratio as for CpMTP, was needed to induce pND1 complexation (Figure 1C). On the other hand, (KH)9 exhibited a higher ability in condensing pND1, as confirmed in Figure 1D. As observed, from a low N/P ratio of 0.1, (KH)9 peptide was able to neutralize the negative charges of the plasmid and, therefore, no band was visible in the agarose gel. This result has been confirmed by determining the complexation capacity (CC) of the (KH)9/pND1 complexes. CC has been calculated from the following equation:

CC (%) = [(pND1)T − (pND1)F/(pND1)T] × 100(1)

where (pND1)T stands for the total amount of pND1 and (pND1)F is the non-bound fraction of pND1 found free in the supernatant.

The CC at the N/P ratio of 0.1 was, approximately, 86%, which was in agreement with the results obtained from agarose gel electrophoresis (Figure 1D). The great capacity of (KH)9 peptide to condense pND1 could be attributed to its high cationic charge density, which is higher than those of WRAP1, WRAP5 or CpMTP (Table 1).

The effect of the addition of the MTS sequence to the peptides did not seem to follow a trend concerning the pND1 complexation. As illustrated in Figure 1E, for MTS-WRAP1, the incorporation of this sequence into the WRAP1 peptide led to an efficient pND1 complexation at lower N/P ratios (3➔1). The opposite effect was, however, observed for WRAP5, where the addition of MTS seemed to hinder the capacity of the peptide for pND1 complexation, as can be seen in Figure 1F (0.5➔1). Moreover, the MTS-(KH)9 peptide greatly promoted pND1 complexation at the investigated N/P ratios (Figure 1G) and this capacity was comparable to the one exhibited by the (KH)9 peptide.

### 3.3. Physicochemical Properties of Peptide/pND1 Complexes

**Figure 2:**
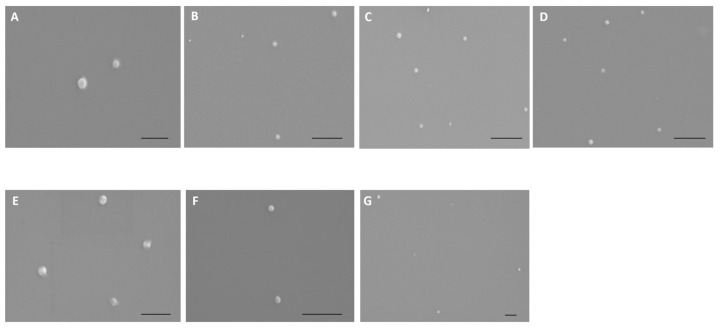
Scanning electron micrographs of CpMTP/pND1 (**A**); WRAP1/pND1 (**B**); WRAP5/pND1 (**C**); (KH)9/pND1 (**D**); MTS-WRAP1/pND1 (**E**); MTS-WRAP5/pND1 (**F**) and MTS-(KH)9/pND1 (**G**) complexes formulated at N/P ratio of 5. Scale bar = 500 nm.

The morphology of peptide/pND1 complexes has been investigated by SEM. Figure 2 showed the obtained images for all complexes formulated at an N/P ratio of 5. All the particles exhibit a spherical or oval shape, and an apparently homogeneous structure. No relevant differences in morphology can be ascribed between peptide/pND1 and MTS-peptide/pND1 systems. Further information on the properties of all the conceived carriers has been evaluated by DLS. The results can be consulted in Table 2. The average size of the formed complexes at different N/P ratios has been determined. The selection of an N/P ratio for each complex studied was based on the ratios that can promote efficient pND1 complexation (Figure 1). All the nano-systems presented sizes below 500 nm, and this parameter was showed to be dependent on the N/P ratio considered at the formulation stage. Independently of the peptide, there was a visible tendency of particle size decrease with the increase of the N/P ratio (**** *p* < 0.0001). The increment of peptide positive charges intensified the interaction with the plasmid, leading to pND1 condensation to a higher extent. This phenomenon creates smaller-sized complexes. Additionally, for all peptide/pND1 particles and for most of the N/P ratios considered, the addition of the MTS sequence to the peptide led to higher sizes (**** *p* < 0.0001 for WRAP1 versus MTS-WRAP1; * *p* < 0.05 for WRAP5 versus MTS-WRAP5; *** *p* < 0.001 for (KH)9 versus MTS-(KH)9). Moreover, the sizes displayed by the developed delivery systems, and particularly, at higher N/P ratios such as an N/P of 5, seemed to be adequate for cellular uptake/internalization purposes. The cellular internalization is clearly influenced by the size, shape, surface charge and surface chemistry of the nano-system, and it may also greatly depend on the cell type [45,46]. In general, spherical complexes displaying a low size (~100–200 nm) are preferentially captured by cells compared to higher-sized carriers (>200 nm). Optimizing the size of nanoparticles can, thus, facilitate both cell uptake and payload delivery into the cells.

A comparative analysis of the size displayed by the carriers, obtained from SEM and DLS, presented some discrepancies in the obtained values. As demonstrated earlier and explained in detail in another publication, this observation can be related to the principles, advantages and limitations of each assay/technique [47].

Furthermore, polydispersity index (PdI), an indication of size distribution, has been also determined. The PdI value may vary from 0.01 for monodisperse particles to 0.5–0.7 for polydisperse ones, and a value higher than 0.7 indicates a broad particle size distribution. The PdI values, listed in Table 2, indicate that peptide/pND1 nanoparticles displaying higher sizes (in the range of 400–500 nm) were polydisperse. Conversely, complexes with smaller sizes were quite monodisperse, exhibiting PdI values in the range of 0.2–0.3, which corresponded to the “classical” PdI values for peptide-based nanoparticles.

Another crucial property of delivery systems is the surface charge they carry. The zeta potential values of the developed particles have been determined by DLS. The obtained values are included in Table 2. As can be observed, peptide/pND1 systems formulated at lower N/P ratios presented negative surface charges. For each peptide under study, an increase in N/P ratio resulted in more positive zeta potential values due to the increment of amine groups in the formulation (**** *p* < 0.0001 for the analysis concerning CpMTP and except for the two lower N/P ratios; **** *p* < 0.0001 for WRAP1; **** *p* < 0.0001 for WRAP5; **** *p* < 0.0001 for the analysis focused on (KH)9 and except for the two lower N/P ratios; **** *p* < 0.0001 for MTS-WRAP1; **** *p* < 0.0001 for MTS-WRAP5; ** *p* < 0.01 for MTS-(KH)9. We also detected a decrease in the positive surface charges of the particles when the MTS sequence was added to the peptides (**** *p* < 0.0001 for WRAP1 versus MTS-WRAP1; *** *p* < 0.0001 for WRAP5 versus MTS-WRAP5; *** *p* < 0.0001 for (KH)9 versus MTS-(KH)9). As shown in Table 2, the magnitude of this effect was more pronounced for MTS-WRAP1/pND1 carriers (**** *p* < 0.0001). Despite this, all high N/P ratio peptide/pND1 nanoparticles possessed positive zeta potential values (+10 mV to +35 mV) that may make them suitable for cellular internalization. Positively charged complexes may interact favorably with the highly anionic sulfated proteoglycan molecules present at the cell surface, promoting the uptake [48,49]. Once inside the cell, targeting specific intracellular organelles can occur due to targeting sequences attached to the translocating peptides.

At this point, it also became clear that the N/P ratio could be used as a tailoring tool to optimize the properties of peptide/pND1 complexes, enhancing its cellular uptake/internalization and, therefore, pND1 delivery.

Based on the above-mentioned arguments, peptide/pND1 complexes formulated at lower N/P ratios were excluded from subsequent studies. In vitro experiments, presented in the next sections, were performed with complexes prepared at N/P ratios of 3 and 5, or only an N/P ratio of 5, due to their adequate sizes, surface charges and higher pDNA CCs. These properties seem to be promising for in vitro applications in the mitochondrial gene therapy field.

### 3.4. Cytotoxic Profile 

**Figure 3:**
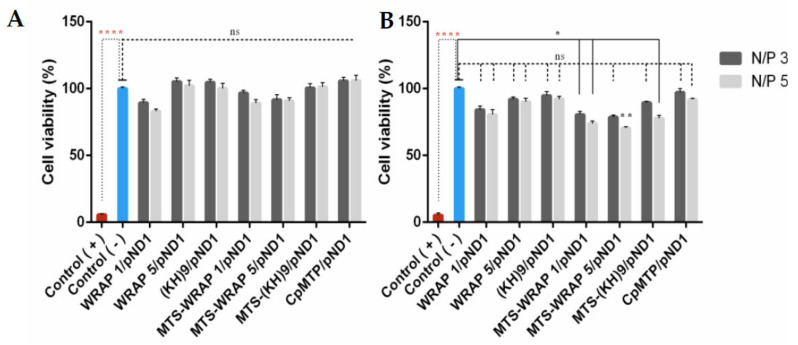
Cellular viability of HeLa cells after 24 h (**A**) and 48 h (**B**) of incubation with WRAP1/pND1, WRAP5/pND1, (KH)9/pND1, MTS-WRAP1/pND1, MTS-WRAP5/pND1, MTS-(KH)9/pND1 and CpMTP/pND1 nanoparticles conceived at N/P ratios of 3 and 5. Non-transfected cells were used as negative control and ethanol-treated cells were used as a positive control. A statistically significant difference was noticed between the positive and negative control (**** *p* < 0.0001). The MTS-WRAP1 N/P ratios of 3 and 5, MTS-(KH)9 N/P ratio of 5 (* *p* ˂ 0.05) and MTS-WRAP5 N/P ratio of 5 (** *p* < 0.01) systems demonstrated a statistical difference in relation to the negative control at 48 h. The viability of cells transfected with the remaining systems did not show statistically significant differences compared to non-transfected cells (ns).

The cytotoxicity of the developed peptide/pND1 complexes has been evaluated, on HeLa cells, by means of an MTT assay. This colorimetric method is useful in assessing the toxicity/safety of drug or gene delivery systems. The cellular viability of HeLa cells has been investigated, at 24 h and 48 h, after incubation with the several nano-systems formulated at N/P ratios of 3 and 5. Non-transfected cells were used as a negative control, and ethanol-treated cells were used as a positive control. The results are shown in Figure 3, A and B corresponding to 24 h and 48 h, respectively. Figure 3A shows that none of the developed carriers induce a cytotoxic effect on the cancer cells at 24 h, with obtained results being statistically not significant (n.s.) in relation to a negative control. A decrease on biocompatibility can be observed, for all peptide/pND1 nanoparticles, at 48 h. In addition, at this time, the effect of the incorporation of the MTS sequence into the peptides seemed to influence the cytotoxic profile displayed by the complexes. MTS-peptide/pND1 carriers exhibited less cellular viability in relation to control, * *p* < 0.05 for MTS-WRAP1/pND1 at both N/P ratios and MTS-(KH)9 at N/P ratio of 5 and ** *p* < 0.01 for MTS-WRAP5/pND1 at N/P of 5. Furthermore, the N/P ratio parameter seemed, additionally, to play a role in the complex´s biocompatibility. Some of the carriers formed at an N/P ratio of 5 induced a decrease in the cellular viability to a higher extent when compared to the effect of complexes conceived at an N/P ratio of 3. This was the case for MTS-WRAP5/pND1 and MTS-(KH)9/pND1. At 48 h, the comparison between the two N/P ratios was statistically significant, MTS-WRAP5/pND1 (*p* = 0.0002) and MTS-(KH)9/pND1 (**** *p* < 0.0001). Despite these observations, all the developed peptide/pND1 complexes showed high cellular viability when tested in HeLa cells and, from the biocompatibility requirement, they could be safely used as gene delivery systems.

### 3.5. Mitochondria Targeting Ability

In the past few years, several research studies have demonstrated efficient mitochondria targeting [19,40,43,44,50,51,52] The efforts in the development of delivery systems displaying this capacity have been fruitful, with various reports on biomolecules or gene delivery into mitochondria [19,39,53,54,55]. Despite this achieved progress, to date, few works have demonstrated effective long-term mitochondrial transgene expression [52]. To contribute to this field, we studied the mitochondria targeting ability of the conceived peptide/pND1 complexes. Our team also searched for relevant differences between these delivery systems to select the most promising complexes for subsequent gene/protein expression studies.

**Figure 4:**
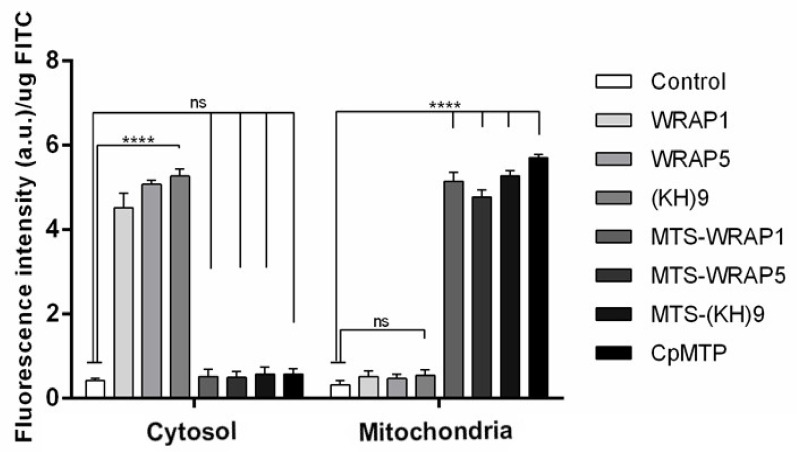
FITC fluorescence intensity (arbitrary units, a.u.) in the cytosol and mitochondria of HeLa cells after 12 h transfection mediated by WRAP1/pND1, WRAP5/pND1, (KH)9/pND1, MTS-WRAP1/pND1, MTS-WRAP5/pND1, MTS-(KH)9/pND1 and CpMTP/pND1 systems conceived at N/P ratio of 5. Untreated cells were used as control. The data were obtained by calculating the average of four independent experiments and are presented as mean ± SD; not significant (n.s.) and **** *p* < 0.0001. A comparison between cytosol and mitochondria for corresponding complexes was performed for all pND1-based systems: not significant for control and **** *p* < 0.0001 for all systems.

**Figure 5:**
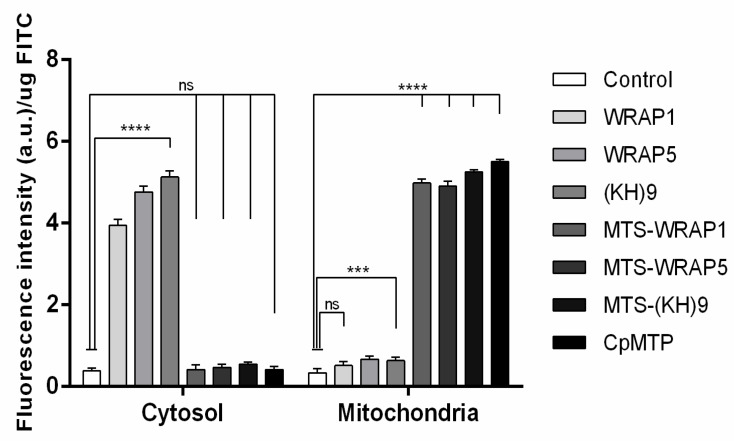
FITC fluorescence intensity (arbitrary units, a.u.) in the cytosol and mitochondria of fibroblast cells after 12h transfection mediated by WRAP1/pND1, WRAP5/pND1, (KH)9/pND1, MTS-WRAP1/pND1, MTS-WRAP5/pND1, MTS-(KH)9/pND1 and CpMTP/pND1 systems conceived at N/P ratio of 5. Untreated cells were used as control. The data were obtained by calculating the average of four independent experiments and are presented as mean ± SD; not significant (n.s.), *** *p* < 0.001 and **** *p* < 0.0001.

The mitochondria targeting capacity of peptide/pND1complexes, formulated at an N/P ratio of 5, was first evaluated by monitoring the FITC-pND1 levels present in the mitochondria, after transfection of HeLa or fibroblast cells. The results of FITC fluorescence intensity, after the separation of mitochondria from the other cellular organelles, are shown in Figure 4 and Figure 5, for HeLa and fibroblast cells, respectively. The data correspond to 12 h transfection mediated by each of the studied peptide/pND1 systems. Untreated cells were used as control. For both cells, the results demonstrated that all the formed carriers have been internalized by the cells and can be found in a particular cellular fraction. The detailed analysis of Figure 4 demonstrated that high levels of stained pND1 were found in the cytosol for the transfection of cancer cells by each MTS-free peptide/pND1 complex (**** *p* < 0.0001 versus control), with WRAP5 and (KH)9 peptide systems exhibiting slightly higher fluorescence levels relative to WRAP1/pND1 particles, * *p* < 0.05. When the transfection is mediated by MTS-peptide and CpMTP/pND1 carriers, a low amount of labeled pND1 is quantified in the cytosolic fraction of HeLa cells (statistically not significant). Our finding is in agreement with other reports on the transfection efficiency of WRAP peptides. Previous studies based on WRAP1- and WRAP5-peptide/siRNA nanoparticles reported on cellular uptake and cytosol delivery, but no mitochondria targeting has been identified [30,36]. This lack of targeting displayed by MTS free peptides was confirmed when mitochondria samples were analyzed. In this organelle, vestigial amounts of FITC-pND1 fluorescence were quantified for the transfection conducted by WRAP1-, WRAP5- or (KH)9 peptide/pND1 complexes (Figure 4). Conversely, when the MTS sequence was incorporated into these peptides, the complexes were able to target mitochondria of HeLa cells as shown by the high fluorescence levels found in this organelle (**** *p* < 0.0001 versus control). As the results suggest, the MTS sequence incorporated into WRAP1, WRAP5 and (KH)9 peptides conferred mitochondrial targeting specificity. It became clear that these peptides per se do not possess this targeting skill. The mitochondria targeting performance of CpMTP peptide was also confirmed, as the transfection by CpMTP/pND1 carriers exhibits higher fluorescence levels (**** *p* < 0.0001). From Figure 4, and between MTS-peptides or CpMTP/pND1 nanoparticles, some differences arose in the obtained FITC-pND1 fluorescence intensity. The transfection mediated by MTS-WRAP5/pND1 systems showed less fluorescence in comparison with the one corresponding to transfection by the complexes based on MTS-(KH)9 and CpMTP peptides, ** *p* < 0.01. This fact may indicate differences in the transfection efficiency and targeting capacity between the developed nano-systems. MTS-(KH)9 and CpMTP peptide/pND1 complexes seemed to present a higher performance in mediating these processes. Similar results were obtained for the study performed on fibroblast cells, as can be observed in Figure 5. A comparison between the two cell lines showed no significant difference, and proved the mitochondrial targeting capacity of the developed MTS-peptide/pND1 and CpMTP/pND1 complexes.

Other authors have reported on the high cell penetration ability of the CpMTP peptide on HeLa cells and on its capacity to efficiently deliver macromolecules to mitochondria. These researchers highlighted the potential application of CpMTP in the transduction and transfection of mitochondria for therapeutics [39].

**Figure 6:**
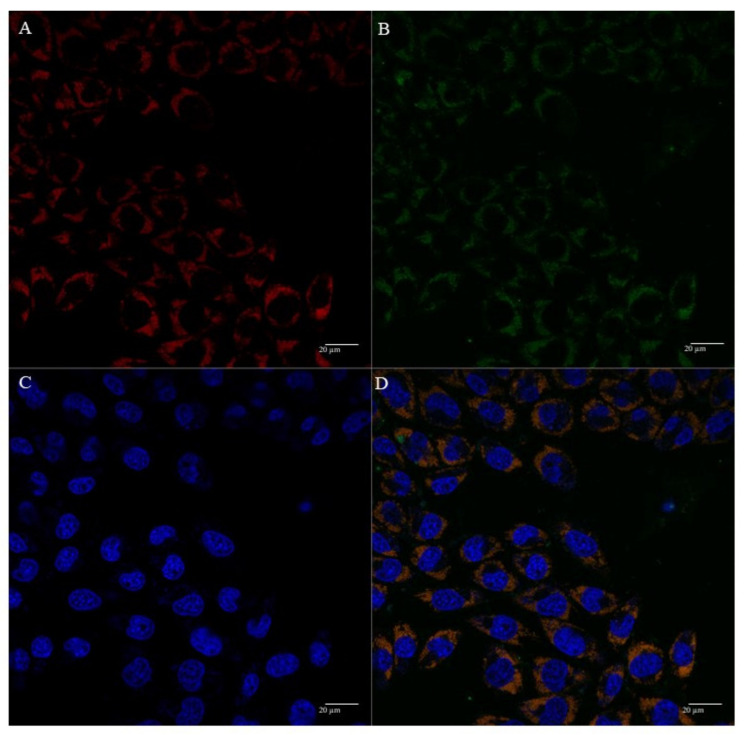
Representative confocal fluorescence image of HeLa cells illustrating the transfection ability and intracellular co-localization of CpMTP/pND1 complexes formulated at an N/P ratio of 5. Mitochondria stained red by MitoTracker (**A**), pND1 green, labeled (**B**), nucleus marked blue by DAPI (**C**), and merged image (**D**). Scale bar = 20 µm.

**Figure 7:**
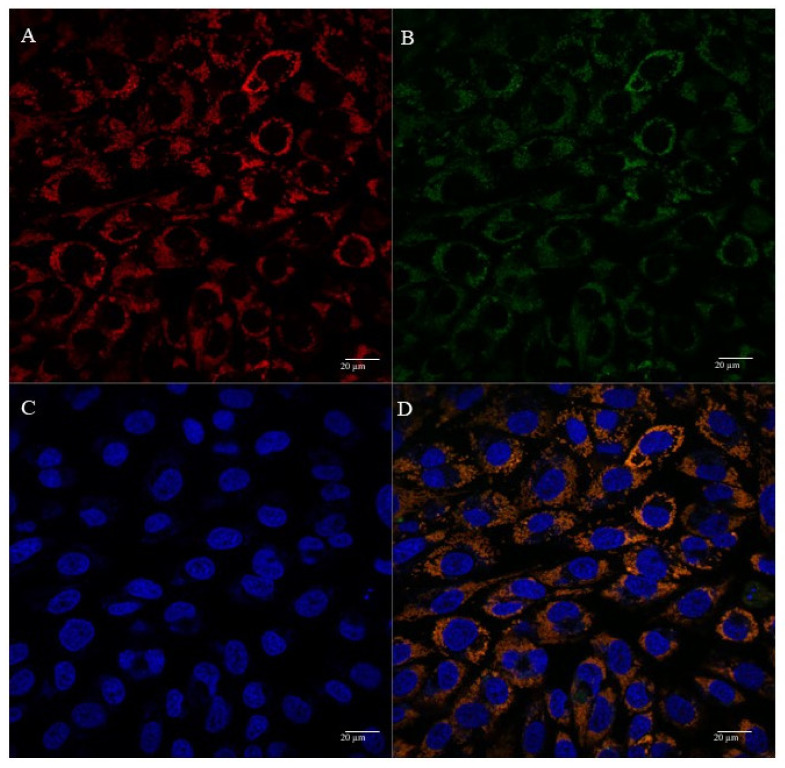
Representative confocal fluorescence image of HeLa cells, illustrating the transfection ability and intracellular co-localization of MTS-WRAP1/pND1 complexes formulated at an N/P ratio of 5. Mitochondria stained red by MitoTracker (**A**), pND1 green, labeled (**B**), nucleus marked blue by DAPI (**C**), and merged image (**D**). Scale bar = 20 µm.

**Figure 8:**
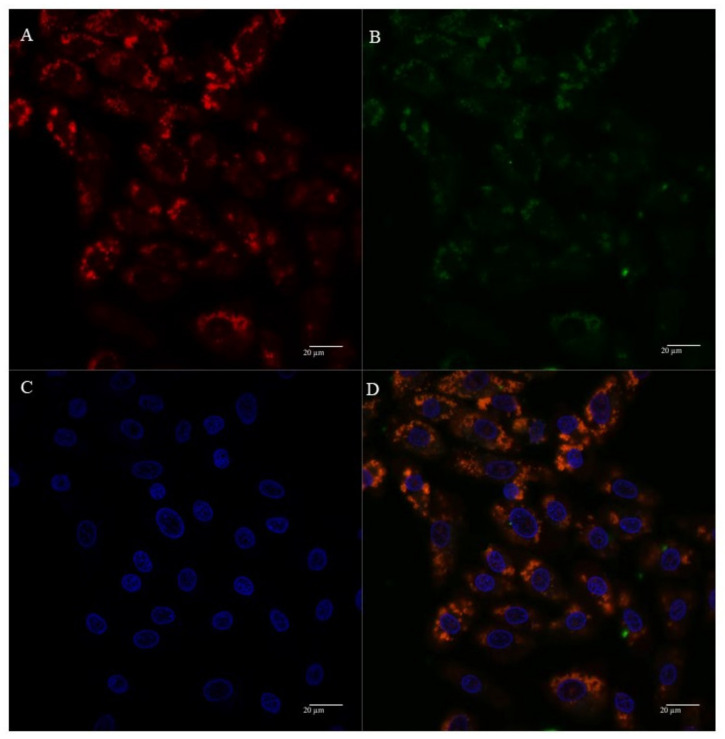
Representative confocal fluorescence image of HeLa cells, illustrating the transfection ability and intracellular co-localization of MTS-WRAP5/pND1 complexes formulated at an N/P ratio of 5. Mitochondria stained red by MitoTracker (**A**), pND1 green, labeled (**B**), nucleus marked blue by DAPI (**C**), and merged image (**D**). Scale bar = 20 µm.

**Figure 9:**
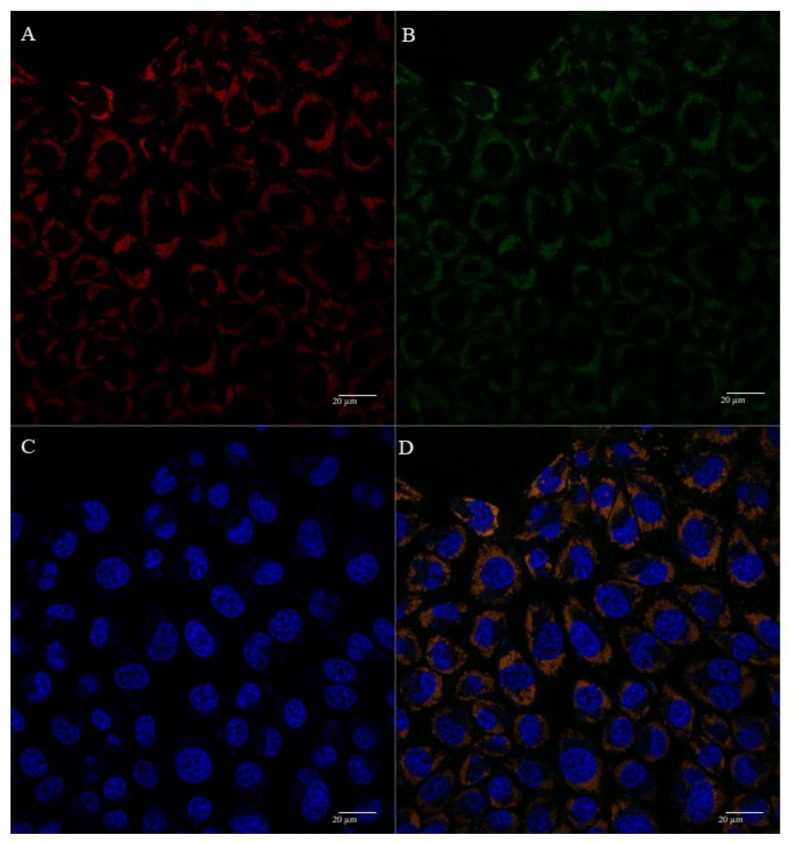
Representative confocal fluorescence image of HeLa cells, illustrating the transfection ability and intracellular co-localization of MTS-(KH)_9_/pND1 complexes formulated at an N/P ratio of 5. Mitochondria stained red by MitoTracker (**A**), pND1 green, labeled (**B**), nucleus marked blue by DAPI (**C**), and merged image (**D**). Scale bar = 20 µm.

The cellular uptake and mitochondrial affinity of MTS-peptide/pND1 and CpMTP/pND1 complexes, prepared at an N/P ratio of 5, have been further investigated by fluorescence confocal microscopy. Real live transfection of cancer cells mediated by the developed carriers has been monitored. DAPI and MitoTracker dyes have been used to stain the nuclei and mitochondria, respectively; pND1 has been labeled with FITC. Images were collected from a series of consecutive Z-planes (Z-stacks, step size of 0.1 µm). Figure 6, Figure 7, Figure 8 and Figure 9 summarize the obtained images, at 6 h of transfection. In all figures, A stands for mitochondria labeled with MitoTracker, B for peptide/FITC-pND1 nanoparticles, C for nuclei stained with DAPI, and D represents the merged image. As can be seen from the green fluorescence on the (B) images of all figures, effective transfection took place and all pND1-loaded carriers were internalized into cells. After cell uptake, the developed complexes were directed to the site of the mitochondrion. Image D, of all the figures, presented a significant accumulation of peptide/pND1 nano-systems into this cellular organelle, as evidenced by the orange color in the merged picture of red-stained mitochondria and green fluorescence from the pDNA in the same field. This microscopy showed that the formulated nanoparticles could be successfully targeted at mitochondria, although at this stage we cannot state if pND1 is, in fact, delivered to mitochondria. The current studies of our research team are focused on the evaluation of pND1 delivery, gene and ND1 protein expression. Hopefully, results on this topic can be reported soon. Here, we undoubtedly proved the mitochondria targeting capacity of the developed complexes. This is a significant step towards mitochondrial gene therapy feasibility.

## 4. Conclusions

The feasibility of mitochondrial gene therapy seeks the conception of a suitable gene delivery system capable of mitochondria targeting. Pursuing this aim, a mitochondrial-targeting sequence has been incorporated into WRAP1, WRAP5 and (KH)9 peptides and, along with the CpMTP peptide, they were used to complex pND1. Instead of studying model cargos, in this work, we approached the concrete reality by using pND1, currently one of the most advanced vectors in the mitochondrial gene therapy field. The formed peptide/pND1 complexes, at various N/P ratios, were revealed to possess adequate physicochemical properties for gene delivery applications, namely, pND1 complexation ability, morphology, size and surface charges. It was found that the N/P ratio could be an effective tool to optimize the characteristics of the developed complexes. Moreover, the new nano-systems did not induce a cytotoxic effect in cancer HeLa cells. An evaluation of mitochondria targeting ability showed the internalization of MTS-peptides/pND1 and CpMTP/pND1 complexes into cells and their targeting to mitochondria. A comparison study revealed differences between the various peptide/pND1 complexes, concerning both their physicochemical properties and targeting skill. Although all MTS-peptides/pND1 complexes offer remarkable properties that can be applied in mitochondrial gene therapy, the ones based on MTS-(KH)9 and CpMTP peptides exhibited a greater capacity to complex pND1, and higher targeting performance. These complexes constitute a promising bet for further research studies on mitochondrial gene/protein expression. The knowledge provided by this report can significantly contribute to optimizing the development of novel peptide-based systems for mitochondrial gene expression.

## Figures and Tables

**Table 1:** List of the synthesized peptides and MTS sequence, including information on peptide sequence, total residues, isotopic mass and positive charges.

Peptide	Peptide Sequence	Total Residues	Isotopic Mass (g/mol)	Positive Charges
MTS	NH2-MLSLRQSIRFFK-CONH2	12	1523.88	3
WRAP1	NH2-LLWRLWRLLWRLWRLL-CONH2	16	2290.42	5
WRAP5	NH2-LLRLLRWWWRLLRLL-CONH2	15	2104.34	5
(KH)9	NH2-KHKHKHKHKHKHKHKHKH-CONH2	18	2403.41	9
CpMTP	NH2-ARLLWLLRGLTLGTAPRRA-CONH2	19	2132.32	4
MTS-WRAP1	NH2-MLSLRQSIRFFK-LLWRLWRLLWRLWRLL-CONH2	28	3797.27	8
MTS-WRAP5	NH2-MLSLRQSIRFFK-LLRLLRWWWRLLRLL-CONH2	27	3611.19	8
MTS-(KH)9	NH2-MLSLRQSIRFFK-KHKHKHKHKHKHKHKHKH-CONH2	30	3910.26	12

**Table 2:** Average zeta potential, mean size and polydispersity index (PdI) for the various peptide/pND1 complexes formulated at various N/P ratios. The values were calculated with the data obtained from six independent measurements (mean ± SD, n = 6), and analyzed by one-way or two-way ANOVA (GraphPad Software version v.8.01, Inc., San Diego, CA, USA). Statistical significance was accepted at a level of * *p* < 0.05.

System	Zeta (mV)	Size (nm)	PdI
CpMTP/pND1 N/P 0.5	−3.50 ± 0.76	492.00 ± 29.58	0.60 ± 0.1
CpMTP/pND1 N/P 1	+2.00 ± 0.58	401.50 ± 25.12	0.52 ± 0.5
CpMTP/pND1 N/P 2	+3.50 ± 0.50	384.50 ± 14.26	0.24 ± 0.04
CpMTP/pND1 N/P 3	+5.83 ± 0.69	313.17 ± 10.34	0.30 ± 0.02
CpMTP/pND1 N/P 5	+12.67 ± 0.75	235.67 ± 12.60	0.21 ± 0.02
WRAP1/pND1 N/P 3	+25.17 ± 0.69	254.17 ± 13.90	0.34 ± 0.05
WRAP1/pND1 N/P 5	+32.67 ± 0.47	161.00 ± 8.82	0.30 ± 0.02
WRAP5/pND1 N/P 0.5	+1.17 ± 0.37	401.00 ± 19.15	0.54 ± 0.1
WRAP5/pND1 N/P 1	+2.00 ± 0.58	388.33 ± 14.75	0.31 ± 0.02
WRAP5/pND1 N/P 2	+10.83 ± 1.21	298.50 ± 12.96	0.33 ± 0.01
WRAP5/pND1 N/P 3	+14.17 ± 0.90	272.33 ± 10.70	0.24 ± 0.02
WRAP5/pND1 N/P 5	+20.67 ± 0.75	186.00 ± 9.82	0.32 ± 0.03
(KH)9/pND1 N/P 0.1	−2.50 ± 0.76	488.17 ± 22.34	0.62 ± 0.05
(KH)9/pND1 N/P 0.2	+1.50 ± 0.50	412.67 ± 18.60	0.53 ± 0.1
(KH)9/pND1 N/P 0.5	+2.67 ± 0.47	403.33 ± 15.75	0.41 ± 0.04
(KH)9/pND1 N/P 1	+4.50 ± 0.50	375.67 ± 14.25	0.24 ± 0.02
(KH)9/pND1 N/P 2	+6.00 ± 0.58	299.67 ± 11.25	0.33 ± 0.02
(KH)9/pND1 N/P 3	+11.83 ± 1.34	260.50 ± 9.76	0.21 ± 0.03
(KH)9/pND1 N/P 5	+22.33 ± 0.75	186.33 ± 9.49	0.34 ± 0.02
MTS-WRAP1/pND1 N/P 1	−1.83 ± 0.90	406.00 ± 18.63	0.42 ± 0.06
MTS-WRAP1/pND1 N/P 2	+1.33 ± 0.75	366.50 ± 14.38	0.30 ± 0.02
MTS-WRAP1/pND1 N/P 3	+6.50 ± 0.76	276.50 ± 13.12	0.21 ± 0.01
MTS-WRAP1/pND1 N/P 5	+11.50 ± 0.76	197.33 ± 8.49	0.20 ± 0.02
MTS-WRAP5/pND1 N/P 1	−2.17 ± 0.90	399.00 ± 12.41	0.44 ± 0.04
MTS-WRAP5/pND1 N/P 2	+7.17 ± 0.90	316.17 ± 10.21	0.27 ± 0.03
MTS-WRAP5/pND1 N/P 3	+10.83 ± 1.07	266.50 ± 8.22	0.31 ± 0.02
MTS-WRAP5/pND1 N/P 5	+19.33 ± 1.60	175.17 ± 10.86	0.32 ± 0.03
MTS-(KH)9/pND1 N/P 0.1	−3.33 ± 0.94	478.50 ± 22.43	0.61 ± 0.05
MTS-(KH)9/pND1 N/P 0.2	−1.00 ± 1.00	463.50 ± 20.63	0.63 ± 0.03
MTS-(KH)9/pND1 N/P 0.5	+1.83 ± 0.37	431.00 ± 18.63	0.52 ± 0.04
MTS-(KH)9/pND1 N/P 1	+3.17 ± 0.69	400.33 ± 14.25	0.44 ± 0.03
MTS-(KH)9/pND1 N/P 2	+5.67 ± 0.75	366.67 ± 11.49	0.30 ± 0.03
MTS-(KH)9/pND1 N/P 3	+8.50 ± 0.50	309.17 ± 9.34	0.22 ± 0.04
MTS-(KH)9/pND1 N/P 5	+14.67 ± 0.75	220.67 ± 9.29	0.24 ± 0.01

## Data Availability

The data presented in this study are available on request from the corresponding author.
